# Supplementary material for: Role of initial facial attractiveness in the perceived aesthetic outcome of convex profile treatment
Source: PeerJ. 2025 Oct 17;13:e19997. doi: 10.7717/peerj.19997 (PMC12536796; doi:10.7717/peerj.19997)
Supplement: Supplemental Information 1 — Detailed statistical analyses related to the evaluation of facial and profile attractiveness and the perceived changes following treatment of convex facial profiles. Tables S1 and S2 summarize attractiveness ratings and pairwise comparisons across rater groups (surgeons, orthodontists, patients, and laypeople). Tables S3 and S5 report ANOVA results testing the effects of initial attractiveness, rater type, and treatment modality (surgery vs. camouflage) on perceived changes in facial and profile appearance. Tables S4 and S6 provide parameter estimates quantifying the influence of these factors on specific facial features (face, lower face, lips, and chin). [file peerj-13-19997-s001.pdf]

PeerJ

Supplementary material

Role of initial facial attractiveness in the perceived aesthetic outcome of convex profile treatment

Supplementary Table 1. Estimated marginal means of facial attractiveness and facial profile attractiveness ratings per rater group.

Dependent Variable	Rater group	Mean	Std. Error	95% Confidence Interval	
				Lower Bound	Upper Bound
Facial attractiveness	Surgeons	36.16	2.39	31.42	40.89
	Orthodontists	32.47	2.39	27.73	37.20
	Patients	40.37	2.39	35.64	45.11
	Laypeople	35.40	2.39	30.67	40.13
Facial profile attractiveness	Surgeons	33.66	2.30	29.10	38.22
	Orthodontists	35.07	2.30	30.52	39.64
	Patients	43.62	2.30	39.06	48.18
	Laypeople	38.62	2.30	34.07	43.18

Supplementary Table 2. Pairwise comparisons per rater group based on estimated marginal means.

Dependent Variable	(I) Rater	(J) Rater	Mean Difference (I-J)	Std. Error	Sig.	95% Confidence Interval for Difference	
						Lower Bound	Upper Bound
Facial attractiveness	Laypeople	Surgeons	-0.76	3.38	0.823	-7.45	5.94
	Laypeople	Orthodontists	2.93	3.38	0.387	-3.76	9.63
	Laypeople	Patients	-4.97	3.38	0.144	-11.67	1.72
	Surgeons	Orthodontists	3.69	3.38	0.277	-3.00	10.39
	Surgeons	Patients	-4.22	3.38	0.215	-10.91	2.48
	Orthodontists	Patients	-7.91*	3.38	0.021	-14.60	-1.21
Facial profile attractiveness	Laypeople	Surgeons	4.96	3.25	0.130	-1.48	11.41
	Laypeople	Orthodontists	3.55	3.25	0.278	-2.90	9.99
	Laypeople	Patients	-4.99	3.25	0.128	-11.44	1.45
	Surgeons	Orthodontists	-1.41	3.26	0.665	-7.86	5.03
	Surgeons	Patients	-9.96*	3.26	0.003	-16.40	-3.51
	Orthodontists	Patients	-8.54*	3.26	0.010	-14.99	-2.09

*The mean difference is significant at the 0.05 level.

Supplementary Table 3. Results of the ANOVAS testing the effect of facial attractiveness, rater type, and treatment group on the assessed changes in facial appearance from pre- to post-treatment condition.

Source	Dependent Variable	df	F	Sig.
Facial attractiveness	Face[a]	1	14.06	<0.001
	Lower Face[b]	1	14.45	<0.001
	Upper Lip[c]	1	4.92	0.028
	Lower Lip[d]	1	6.60	0.011
	Chin[e]	1	10.79	0.001
Treatment group	Face	1	42.98	<0.001
	Lower Face	1	56.13	<0.001
	Upper Lip	1	37.22	<0.001
	Lower Lip	1	40.94	<0.001
	Chin	1	35.72	<0.001
Rater type	Face	3	0.54	0.656
	Lower Face	3	1.00	0.397
	Upper Lip	3	0.24	0.866
	Lower Lip	3	1.39	0.249
	Chin	3	1.38	0.252
Treatment group * Rater type	Face	3	0.12	0.948
	Lower Face	3	0.11	0.953
	Upper Lip	3	0.22	0.879
	Lower Lip	3	0.01	0.999
	Chin	3	0.24	0.868

[a]R Squared = 0.38 (Adjusted R Squared = 0.34), [b]R Squared = 0.43 (Adjusted R Squared = 0.40), [c]R Squared = 0.29 (Adjusted R Squared = 0.25), [d]R Squared = 0.34 (Adjusted R Squared = 0.30), [e]R Squared = 0.34 (Adjusted R Squared = 0.30). df: degrees of freedom. F: F-value. Sig.: Significance shown as p-values.

Supplementary Table 4. Parameter estimates indicating the effect of tested factors on the perceived changes in facial appearance due to treatment (dependent variables).

Dependent Variable	Parameter	B	95% Confidence Interval		Sig.
			Lower Bound	Upper Bound	
Face	Intercept	74.22	66.21	82.24	<0.001
	Facial attractiveness	-0.29	-0.44	-0.14	<0.001
	Camouflage (ref.: Surgery)	-12.08	-20.35	-3.80	0.005
	Laypeople (ref.: Patients)	1.37	-6.87	9.62	0.743
	Surgeons (ref.: Patients)	2.87	-5.37	11.11	0.493
	Orthodontists (ref.: Patients)	4.79	-3.57	13.14	0.260
Lower Face	Intercept	77.58	69.31	85.86	<0.001
	Facial attractiveness	-0.30	-0.45	-0.14	<0.001
	Camouflage (ref.: Surgery)	-17.55	-26.10	-9.01	<0.001
	Laypeople (ref.: Patients)	-2.11	-10.63	6.40	0.624
	Surgeons (ref.: Patients)	1.17	-7.34	9.68	0.787
	Orthodontists (ref.: Patients)	4.11	-4.52	12.74	0.348
Upper Lip	Intercept	70.89	62.78	79.00	<0.001
	Facial attractiveness	-0.17	-0.32	-0.02	0.028
	Camouflage (ref.: Surgery)	-14.50	-22.88	-6.12	<0.001
	Laypeople (ref.: Patients)	-2.11	-10.45	6.24	0.618
	Surgeons (ref.: Patients)	-2.63	-10.97	5.71	0.534
	Orthodontists (ref.: Patients)	-1.77	-10.23	6.70	0.681
Lower Lip	Intercept	74.04	65.17	82.92	<0.001
	Facial attractiveness	-0.22	-0.38	-0.05	0.011
	Camouflage (ref.: Surgery)	-15.55	-24.72	-6.39	0.001
	Laypeople (ref.: Patients)	-1.77	-10.90	7.36	0.702
	Surgeons (ref.: Patients)	3.15	-5.98	12.27	0.496
	Orthodontists (ref.: Patients)	4.10	-5.15	13.36	0.382
Chin	Intercept	75.84	66.69	85.00	<0.001
	Facial attractiveness	-0.29	-0.46	-0.11	0.001
	Camouflage (ref.: Surgery)	-14.44	-23.90	-4.99	0.003
	Laypeople (ref.: Patients)	-2.40	-11.83	7.02	0.615
	Surgeons (ref.: Patients)	5.36	-4.06	14.78	0.262
	Orthodontists (ref.: Patients)	5.21	-4.35	14.76	0.283

Supplementary Table 5. Results of the ANOVAS testing the effect of facial profile attractiveness, rater type, and treatment group on the assessed changes in facial profile appearance from pre- to post-treatment condition.

Source	Dependent Variable	df	F	Sig.
Facial profile attractiveness	Face[a]	1	8.60	0.004
	Lower Face[b]	1	5.16	0.025
	Upper Lip[c]	1	3.03	0.084
	Lower Lip[d]	1	2.89	0.091
	Chin[e]	1	3.73	0.056
Treatment group	Face	1	71.81	<0.001
	Lower Face	1	64.64	<0.001
	Upper Lip	1	40.84	<0.001
	Lower Lip	1	41.91	<0.001
	Chin	1	65.51	<0.001
Rater type	Face	3	1.07	0.363
	Lower Face	3	0.36	0.784
	Upper Lip	3	0.72	0.544
	Lower Lip	3	1.47	0.225
	Chin	3	0.88	0.456
Treatment group * Rater type	Face	3	0.35	0.790
	Lower Face	3	0.36	0.780
	Upper Lip	3	0.20	0.899
	Lower Lip	3	0.45	0.721
	Chin	3	1.02	0.387

[a]R Squared = 0.46 (Adjusted R Squared = 0.42), [b]R Squared = 0.41 (Adjusted R Squared = 0.38), [c]R Squared = 0.30 (Adjusted R Squared = 0.26), [d]R Squared = 0.33 (Adjusted R Squared = 0.29), [e]R Squared = 0.41 (Adjusted R Squared = 0.38)

Supplementary Table 6. Parameter estimates indicating the effect of tested factors on the perceived changes in facial profile appearance due to treatment (dependent variables).

Dependent Variable	Parameter	B	95% Confidence Interval		Sig.
			Lower Bound	Upper Bound	
Face	Intercept	74.46	67.19	81.73	<0.001
	Facial profile attractiveness	-0.20	-0.33	-0.06	0.004
	Camouflage (ref.: Surgery)	-12.91	-19.93	-5.89	<0.001
	Laypeople (ref.: Patients)	-1.42	-8.42	5.57	0.688
	Surgeons (ref.: Patients)	1.90	-5.23	9.03	0.599
	Orthodontists (ref.: Patients)	3.95	-3.21	11.11	0.278
Lower Face	Intercept	73.92	66.07	81.77	<0.001
	Facial profile attractiveness	-0.16	-0.31	-0.02	0.025
	Camouflage (ref.: Surgery)	-12.70	-20.28	-5.12	0.001
	Laypeople (ref.: Patients)	0.47	-7.08	8.02	0.903
	Surgeons (ref.: Patients)	2.27	-5.43	9.96	0.561
	Orthodontists (ref.: Patients)	3.10	-4.64	10.83	0.430
Upper Lip	Intercept	66.45	58.48	74.42	<0.001
	Facial profile attractiveness	-0.13	-0.27	0.02	0.084
	Camouflage (ref.: Surgery)	-11.61	-19.30	-3.91	0.003
	Laypeople (ref.: Patients)	2.32	-5.35	9.98	0.551
	Surgeons (ref.: Patients)	1.59	-6.22	9.40	0.688
	Orthodontists (ref.: Patients)	-2.94	-10.79	4.91	0.461
Lower Lip	Intercept	70.01	61.22	78.80	<0.001
	Facial profile attractiveness	-0.14	-0.30	0.02	0.091
	Camouflage (ref.: Surgery)	-11.26	-19.75	-2.77	0.010
	Laypeople (ref.: Patients)	0.12	-8.34	8.58	0.978
	Surgeons (ref.: Patients)	4.96	-3.66	13.58	0.257
	Orthodontists (ref.: Patients)	7.15	-1.51	15.81	0.105
Chin	Intercept	74.33	66.27	82.40	<0.001
	Facial profile attractiveness	-0.14	-0.29	0.00	0.056
	Camouflage (ref.: Surgery)	-12.26	-20.05	-4.48	0.002
	Laypeople (ref.: Patients)	-2.34	-10.10	5.42	0.553
	Surgeons (ref.: Patients)	2.98	-4.92	10.89	0.457
	Orthodontists (ref.: Patients)	4.30	-3.65	12.24	0.287
